# Reliability and Validity of Korean Version of Crohn's and Ulcerative Colitis Questionnaire-8

**DOI:** 10.1155/2022/9746899

**Published:** 2022-10-07

**Authors:** Sung-Goo Kang, Tae-Geun Gweon, Han Hee Lee, Kang-Moon Lee, Sung Hoon Jung, Sang-Bum Kang

**Affiliations:** ^1^Department of Family Medicine, St. Vincent's Hospital, College of Medicine, The Catholic University of Korea, Republic of Korea; ^2^Department of Internal Medicine, College of Medicine, The Catholic University of Korea, Republic of Korea

## Abstract

**Background:**

Patients with inflammatory bowel disease (IBD) have a decreased quality of life (QoL), the improvement of which is a treatment goal. The CUCQ-8 is a verified simple and effective QoL measurement tool. We validated the Korean version of CUCQ-8 with the approval of its developer.

**Methods:**

We investigated the correlation between the Korean version of CUCQ-8 and the IBDQ-32 in patients with IBD.

**Results:**

In all, 147 subjects (male, 97 (66.0%); female, 50 (34.0%); mean age 36.2 ± 13.5 years) were analyzed. Cronbach's alpha coefficient of the CUCQ-8 was 0.833, indicating very high internal consistency. The Korean version of the CUCQ-8 showed a significant correlation with the IBDQ-32 and its subscales (correlation coefficient, >0.75).

**Conclusions:**

The Korean version of the CUCQ-8 has high reliability and construct validity and can be used to evaluate the QoL of patients with IBD.

## 1. Introduction

Inflammatory bowel diseases (IBDs), such as Crohn's disease (CD) and ulcerative colitis (UC), are idiopathic chronic inflammatory diseases of the gastrointestinal tract [1–3]. Globally, the number of patients with IBD continues to increase, mainly in developing countries, and the incidence and prevalence of IBD are steadily increasing in South Korea [4]. The main symptoms of IBD are abdominal pain, diarrhea, bloody stool, and urgency, and extraintestinal symptoms in the joints and skin. IBD is incurable, recurs repeatedly, and requires hospitalization in some cases. In addition, the physical symptoms of IBD can cause chronic fatigue and depression, which are related to a decrease in the quality of life (QoL) [5–8].

Demographic characteristics, disease-related factors, and psychosocial factors are related to the QoL of patients with IBD. A decreased QoL is related to disease activity, Crohn's disease, female sex, older age, and IBD surgery [9].

QoL measures provide insight into patients' perceptions of their health and how it is affected by the treatments they receive. Therefore, it is important to measure the QoL of patients with IBD, for which a simple and reliable tool is required.

The Inflammatory Bowel Disease Questionnaire (IBDQ) is used for research on the QoL of patients with IBD. In South Korea, a study evaluated the QoL of patients with Crohn's disease, ulcerative colitis, and Behcet's disease using the IBDQ [10]. However, the IBDQ is complicated and inconvenient to use in clinical practice because of the large number of questions and high level of complexity. The Crohn's and Ulcerative Colitis Questionnaire-8 (CUCQ-8) is a verified, simple, and effective QoL measurement tool [11]. To date, the CUCQ-8 has not been approved by its developer for use in South Korea. With the approval of the developer, we evaluated the reliability, validity, and utility of the Korean version of the CUCQ-8 for measuring the QoL of patients with IBD in South Korea.

## 2. Materials and Methods

### 2.1. Study Population

This was a multicenter study involving five institutions of the Catholic University of Korea (Daejeon St. Mary's, Eunpyeong St. Mary's, Yeouido St. Mary's, Bucheon St. Mary's, and St. Vincent's Hospitals) and including 147 patients diagnosed with Crohn's disease or ulcerative colitis. Consecutive patients with confirmed diagnoses of CD and UC were enrolled prospectively. We excluded patients who were under 18, non-Korean speakers, and with severe mental illness. Disease activity was measured using the Crohn's Disease Activity Index (CDAI) [12] and Mayo score [13].

The researchers explained the purpose of the study and the composition, contents, and precautions of the questionnaire to the subjects. The subjects were given sufficient time to read and respond to the questions. Subjects completed the questionnaire and asked the researchers to clarify any ambiguity. The subjects' age, sex, body mass index (BMI), disease duration, and history of IBD surgery were investigated.

### 2.2. IBDQ

The IBDQ, developed by Guyatt et al. [14], is used to evaluate the QoL of patients with IBD. The IBDQ consists of 32 items and has four subscales. There are 10 items on bowel symptoms, 5 on systemic symptoms, 5 on social functions, and 12 on emotional functions. Each item is rated on a 7-point Likert scale, with 1 indicating the most severe problem and 7 indicating no problem at all. We received permission from the McMaster Industry Liaison Office, which holds the copyright for the IBDQ, to use the Korean version of the IBDQ. The total IBDQ score is 32 to 224 points, and a higher score reflects a higher QoL.

### 2.3. CUCQ-8 Scoring

The CUCU-8 questionnaire evaluates intestinal problems in the prior 2 weeks and their effects on QoL. It assesses loose or runny bowel movement, bloody stool, tiredness, frustration, urgency or awakening at night to use the toilet, and social inhibition. The total score is 0 to 24, and the higher the score, the lower the QoL.

### 2.4. Translation of the CUCQ-8

The CUCQ-8 items were translated with the approval of the developer, Laith Al-Rubaiy, and according to the recommendations of Guillemin et al. [15] and Beaton et al. [15, 16] First, two people (one the corresponding author) fluent in both English and Korean with Korean as their mother tongue translated the items from English to Korean. One translator was blinded to the purpose of the study to improve the quality of the translation. Next, four specialists discussed and analyzed the differences between the two translations and generated a complete translation. Subsequently, two new translators (who were not involved in biomedical science and unaware of the purpose of the study) back-translated the items into English. Finally, four specialists discussed and analyzed the reverse translation to produce the final Korean version of the CUCQ-8.

### 2.5. Statistical Analysis

The Statistical Package for the Social Sciences (SPSS) version 18.0 for Windows was used for statistical analysis. The CUCQ-8 items were assessed for internal consistency using Cronbach's alpha reliability coefficient [17]. The convergent validity of the total CUCQ-8 score was tested using Pearson's correlation coefficient with the IBDQ score. Chi-square and *t*-tests were performed to examine differences in variables between the IBD groups, and multiple linear regression tests were conducted to identify factors affecting the CUCQ-8 score. Exploratory factor analysis was performed to assess the structure of the CUCQ-8 questionnaire. Factors with eigenvalues of >1 were identified by principal component analysis. A value of *P* < 0.05 was taken to indicate statistical significance.

### 2.6. Ethical Considerations

This study was conducted in accordance with the Declaration of Helsinki and was approved by the institutional review boards of the participating hospitals (XC19QEDI0052). Written informed consent was obtained from the subjects. Subjects were instructed to respond to all questionnaire items after reading the instructions.

## 3. Results

### 3.1. Demographics and Clinical Characteristics

In all, 147 subjects of mean age 36.2 ± 13.5 years were included. All enrolled patients were Asian. Among them, 50 (34.0%) were female and 97 (66.0%) were male. Although the UC group was significantly older than the CD group, there were no significant differences in sex ratio, BMI, or disease duration. There were no significant differences in the CUCQ-8 total score or IBDQ total score between the UC and CD groups (Table 1). The CUCQ total score according to demographic characteristics did not differ significantly between the UC and CD groups (Table 2).

### 3.2. Internal Consistency and Structural Validity

Cronbach's alpha coefficient of CUCQ-8 was 0.833, indicating very high internal consistency. To investigate the structure validity of the CUCQ-8, principal component analysis was performed after varimax rotation. Two factors with eigenvalues of >1.0 explained 63.0% of the variables. Factor 1 (eigenvalue 3.9) explained 35.7% of the variables, including five CUCQ-8 items. The factor loading of all-but-one item exceeded 0.60. Factor 2 (eigenvalue 1.1) explained 27.3% of the variables, including three CUCQ-8 items. The factor loading of all-but-one item of factor 2 exceeded 0.60.

### 3.3. Construct Validity

**Figure 1:**
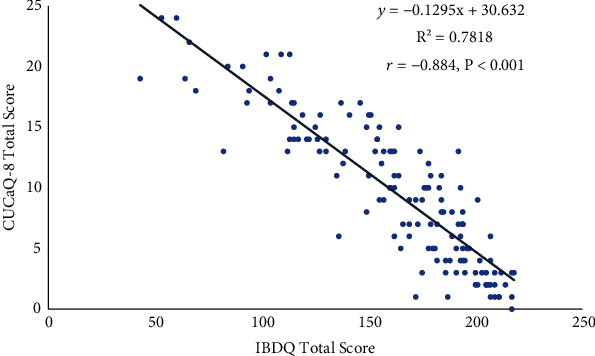
Correlation between CUCQ-8 total score and IBDQ total score.

The CUCQ-8 had good construct validity. The Korean CUCQ-8 total score showed a significant correlation with the IBDQ total score and its subscales. The correlation coefficient for the relationships between the four subtypes of the IBDQ and the CDCQ-8 total score was very high at ≥0.75, showing statistical significance (Table 3 and Figure 1). The Mayo and CDAI scores were significantly positively correlated with the CUCQ-8 total score.

### 3.4. Factors Affecting the CUCQ-8 Total Score

Multiple linear regressions showed that age, sex, BMI, and disease duration did not affect the CUCQ-8 total score in patients with UC or CD. Disease activity (Mayo score for UC and CDAI for CD) had a statistically significant effect on the CUCQ-8 total score (Tables 4 and 5).

## 4. Discussion

QoL measures provide insight into patients' perceptions of their health and how it is affected by the treatments they receive. Therefore, it is important to measure the QoL of patients with IBD. CUCQ-32 and IBDQ-32 have been used to measure quality of life in IBD [10, 11, 17]. However, these tools are very complex and time-consuming. CUCQ-8 was recently validated as a reliable and suitable short questionnaire for monitoring QOL in IBD patients similar to CUCQ-32. While IBDQ-32 should be used for a fee, CUCQ-8 is available free for healthcare providers to support patient care without licensing fees in clinical practice to assess QoL. Therefore, we evaluated the reliability and validity of the Korean version of the CUCQ-8, a measure of the QoL of patients with IBD.

Cronbach's alpha value of >0.7 is considered reliable [15]. In the first study of the CUCQ-8 [11], Cronbach's alpha coefficient was 0.84 in patients with mild to moderate IBD. Hutchings et al. [18] reported that in patients with severe UC, the CUCQ-8 had Cronbach's alpha coefficient of 0.845, indicating excellent internal consistency. In this study, Cronbach's alpha coefficient of the Korean version of the CUCQ-8 was also very good at 0.833.

Principal component analysis detected two major factors with eigenvalues of >1, explaining 63.0% of the total variance in the CUCQ-8. The first factor explained 35.7% of the total variance, and the eigenvalue was 3.96. The items included in the first factor were “1. On how many days over the last 2 weeks have you felt tired?”, “2. In the last 2 weeks did your bowel condition prevent you from going out socially?”, “3. On how many days over the last 2 weeks have you felt generally unwell?”, “4. On how many days over the last 2 weeks have you felt pain in your abdomen?”, and “6. On how many days over the last 2 weeks has your abdomen felt bloated?”. The first factor consisted of five items, including abdominal pain or discomfort symptoms and related fatigue and an unhealthy feeling. The second factor explained 27.3% of the total variance, and the eigenvalue was 1.1. The items included in this factor were “5. On how many nights in the last 2 weeks have you had to get up to use the toilet because of your bowel condition after you have gone to bed?”, “7. In the last 2 weeks have you felt upset?”, and “8. On how many days over the last 2 weeks have you had to rush to the toilet?”. The second factor consisted of a total of three items including urge to defecate and mainly consisted of items such as emotion related to this symptom.

The total score on the Korean CUCQ-8 had very good construct validity and was significantly correlated with the IBDQ total score. The CUCQ-8 total score had correlation coefficients of ≥0.75 with not only the IBDQ total score but also its four subscales.

This study had several limitations. First, there were fewer patients with CD than with UC. However, compared to previous similar studies (*n* = 150–200), the total number of subjects was not small [10, 11]. Second, few of the subjects had severe IBD. In the future, additional studies on acute severe patients are needed. Finally, educational background, economic background, and marital status, which affect QoL, were not investigated.

However, despite these limitations, our findings show that the Korean version of the CUCQ-8 is suitable for measuring the QoL of patients with IBD. The IBDQ has 32 questions and takes a long time to complete and for the total score to be calculated. In comparison, the Korean version of the CUCQ-8 has just eight questions and takes 1–2 min to complete. In addition, because each item can be converted into a 4-point Likert scale, the total score can be calculated relatively quickly. In addition, as mentioned above, the Pearson correlation coefficient of the CUCQ-8 total score and IBDQ total score in this study was 0.884. Given that the Pearson correlation coefficient of the CUCQ-8 and CUCQ-32 total score was originally reported to be 0.91 [11], the CUCQ-8 has very good construct validity. These data demonstrate the utility of the Korean version of CUCQ-8 for measuring the QoL of patients with IBD.

A follow-up study is needed to determine whether the QoL is improved when disease activity is reduced. In addition, the Korean version of the CUCQ-8 will be useful for evaluating the efficacy of novel biologic therapeutics for IBD.

## Figures and Tables

**Table 1:** Baseline characteristics of the study population.

	IBD total (*n* = 147)	UC (*n* = 97)	CD (*n* = 50)	*P* value
Age (years)	36.2 ± 13.5	40.3 ± 13.8	28.3 ± 8.70	<0.001
Sex				0.070
Male	97 (66.0)	59 (60.8)	38 (76.0)	
Female	50 (34.0)	38 (39.2)	12 (24.0)	
BMI (kg/m^2^)	22.6 ± 3.7	22.7 ± 3.3	22.3 ± 3.5	0.594
Duration of disease (years)	4.6 ± 5.9	4.6 ± 5.9	4.7 ± 6.0	0.921
≤1year	42 (28.6)	25 (25.8)	17 (34.0)	
1-5 years	59 (40.1)	42 (43.3)	17 (34.0)	
≥5 years	46 (31.3)	30 (30.9)	16 (32.0)	
History of operation for IBD	11 (7.5)	0 (0)	11 (22)	<0.001
CUCQ-8 total score	9.7 ± 5.9	9.3 ± 6.0	10.4 ± 5.8	0.297
IBDQ total score	161.2 ± 39.9	162.7 ± 40.5	158.2 ± 39.0	0.521
IBDQ-BS	52.0 ± 13.3	52.0 ± 13.8	51.9 ± 12.4	0.959
IBDQ-SS	23.2 ± 6.7	23.8 ± 6.5	21.9 ± 7.0	0.096
IBDQ-EF	58.5 ± 15.6	59.3 ± 15.4	56.8 ± 16.1	0.344
IBDQ-SF	27.6 ± 7.5	27.5 ± 7.9	27.7 ± 6.6	0.900

Data are mean ± SD or *N* (%). *P* values were obtained by independent *t*-tests or chi-square tests. Abbreviations: BMI: body mass index; CUCQ-8: Crohn's and Ulcerative Colitis Questionnaire-8; IBDQ: Inflammatory Bowel Disease Questionnaire; BS: bowel symptoms; SS: social symptoms; EF: emotional function; SF: social function.

**Table 2:** CUCQ-8 total score according to demographic characteristics.

	IBD total (*n* = 147)	UC (*n* = 97)	CD (*n* = 50)	*P* value
Age (years)				
<40	9.5 ± 5.9	9.1 ± 6.1	10.0 ± 5.8	0.493
≥40	10.1 ± 6.0	9.6 ± 6.0	13.0 ± 5.6	0.169
Sex				
Male	9.6 ± 6.2	9.3 ± 6.4	10.0 ± 5.9	0.626
Female	9.9 ± 5.4	9.2 ± 5.3	11.8 ± 5.5	0.168
BMI (kg/m^2^)				
<25	9.9 ± 6.1	9.7 ± 6.1	10.4 ± 6.0	0.570
≥25	8.0 ± 4.9	7.2 ± 5.3	9.4 ± 4.1	0.273
Duration of disease (years)				
<1year	11.1 ± 6.0	11.0 ± 6.4	11.2 ± 5.6	0.945
1-5 years	8.8 ± 6.2	8.6 ± 6.4	9.3 ± 6.0	0.736
>5 years	9.4 ± 5.3	8.7 ± 4.8	10.6 ± 5.9	0.256
History of operation for IBD				
No	9.6 ± 5.9	9.3 ± 6.0	10.2 ± 5.8	0.411
Yes	11.0 ± 5.8	—	11.0 ± 5.8	—

Data are mean ± SD or *N* (%). *P* values were obtained by independent *t*-tests or chi-square tests. Abbreviations: BMI: body mass index; CUCQ-8: Crohn's and Ulcerative Colitis Questionnaire-8.

**Table 3:** Correlation between CUCQ-8 total score and IBDQ total and subscales score in UC and CD.

	IBD total		UC		CD	
	*r*	*P* value	*r*	*P* value	*r*	*P* value
IBDQ total score	-0.884	<0.001	-0.889	<0.001	-0.874	<0.001
IBDQ-BS	-0.848	<0.001	-0.857	<0.001	-0.837	<0.001
IBDQ-SS	-0.829	<0.001	-0.845	<0.001	-0.796	<0.001
IBDQ-EF	-0.821	<0.001	-0.828	<0.001	-0.805	<0.001
IBDQ-SF	-0.763	<0.001	-0.760	<0.001	-0.784	<0.001
Mayo score			0.438	<0.001		
CDAI					0.425	0.003

*P* value was calculated by Pearson's correlation. Abbreviations: CUCQ-8: Crohn's and Ulcerative Colitis Questionnaire-8; IBDQ: Inflammatory Bowel Disease Questionnaire; BS: bowel symptoms; SS: social symptoms; EF: emotional function; SF: social function; CDAI: Crohn's Disease Activity Index.

**Table 4:** Multiple linear regression between CUCQ-8 total score and other cofactors in UC patients.

	*β*	*P* value
Age (years)	0.030	0.484
Sex (female)	0.129	0.920
BMI (kg/m^2^)	-0.291	0.135
Disease duration (years)	-0.111	0.277
Mayo score	0.847	<0.001

Abbreviations: BMI: body mass index; CUCQ-8: Crohn's and Ulcerative Colitis Questionnaire-8.

**Table 5:** Multiple linear regression between CUCQ-8 total score and other cofactors in CD patients.

	*β*	*P* value
Age (years)	-0.046	0.760
Sex (female)	2.415	0.213
BMI (kg/m^2^)	0.055	0.767
Disease duration (years)	0.148	0.508
History of operation for IBD	1.289	0.583
CDAI	2.756	0.003

Abbreviations: BMI: body mass index; CUCQ-8: Crohn's and Ulcerative Colitis Questionnaire-8; CDAI: Crohn's Disease Activity Index.

## Data Availability

The datasets generated and/or analyzed during the current study are available from the corresponding author on reasonable request.

## Supplementary Materials

Supplementary Materials English Certificate.pdf.
