# Supplementary material for: Reliability and Validity of Korean Version of Crohn's and Ulcerative Colitis Questionnaire-8
Source: Biomed Res Int. 2022 Oct 7;2022:9746899. doi: 10.1155/2022/9746899 (PMC9568317; doi:10.1155/2022/9746899)
Supplement: Supplementary Materials — English Certificate.pdf. [file 9746899.f1.pdf]

Textcheck Certificate

Refnum:	22032515
Title:	Reliability and Validity of Korean version of Crohn's and Ulcerative Colitis Questionnaire-8
Date:	2022/03/30

We hereby certify that Textcheck has checked and corrected the English in the manuscript named above.

A specialist editor with suitable professional knowledge (M.Sc. or Ph.D./M.D.) reviewed and corrected the English. An English language specialist subsequently checked the paper again. The first language of both editors is English.

Please direct any questions regarding this certificate or the English in the certified paper to: certified@textcheck.com
(Please quote our reference number: '22032515')

The English in this document has been checked by at least two professional editors, both native speakers of English. For a certificate, please see:

http://www.textcheck.com/certificate/zqXRpO

The above statement is here to inform reviewers—who may not be native speakers of English—that the English in this document has been professionally checked. If the link to the certificate above is deleted and copied into a letter then the reviewers will not see it.

We STRONGLY recommend that no changes are made. Textcheck should be the last step before final formatting. Typically, authors' changes result in errors in the English, not improvements.

If any text has been misunderstood then please REWRITE the sentence(s) that you need to change, using this file, SAVE, and upload a clean (unmarked) final version of the document to: http://www.textcheck.com/client/submit for a final check. Please do not mark your changes (no highlighting, colored font, bold, etc.) and do not include notes, questions, or comments. Changes that are not because of misunderstanding may incur additional charges; please see: http://www.textcheck.com/text/page/revisions. Requests for a final check should be made within 1 month. For more detailed information, please see: 'When you receive your completed document' at http://www.textcheck.com/text/page/guidelines.

REVISED DOCUMENTS: A previously checked document that needs changed and new sentences checked (such as post review) is termed a 'Revised Document' (http://www.textcheck.com/text/page/fees). Revised Documents should be uploaded via 'Submit Document' in your online account, with a note that the file is a revision of '22032515'. Please do not mark your changes; we will use MS Word to compare the document with the most recent complete previous version in your account. When doing so, we cannot consider extracts or versions earlier than the most recent previous version. It is therefore important to upload complete documents. The fee for a Revised Document is based on the wordcount of ALL new and changed sentences. We do not accept new or revised documents on the basis of requests to 'check only marked text'.
